# Comment on “Evolutionary transitions between beneficial and phytopathogenic *Rhodococcus* challenge disease management”

**DOI:** 10.7554/eLife.35238

**Published:** 2018-05-08

**Authors:** Danny Vereecke

**Affiliations:** 1 Department of Applied BiosciencesGhent UniversityGhentBelgium; University of ChicagoUnited States; Max Planck Institute for Developmental BiologyGermany

**Keywords:** *Rhodococcus fascians*, *Rhodococcus corynebacterioides*, leafy gall syndrome, pistachio bushy top syndrome, Other

## Abstract

I would like to report significant issues of concern regarding this paper (Savory et al., 2017).

## Evidence that nonpathogenic isolates are benign/mutualistic

**Figure 1.**
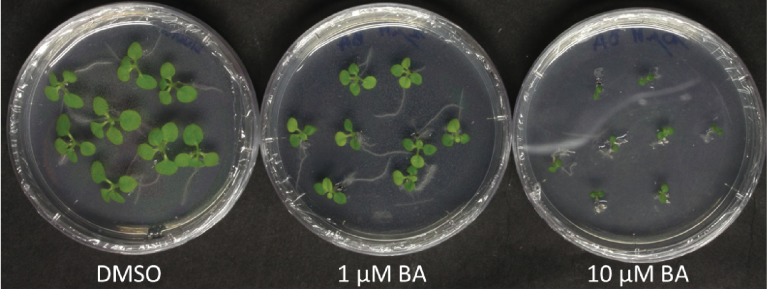
Effect of 1 µM and 10 µM BA on shoot development of *N. tabacum* W38 (12 days after transfer) using the same procedure as Savory et al. (Savory et al., 2017), with the exception that the plates were not grown vertically. Note the excessive number of root hairs in two of the plants in the plate with 1 µM BA; these roots are growing on the medium.

First of all, *Rhodococcus fascians* is defined by its effect on shoots, so it is peculiar that in this work root development was chosen as a phenotype. The alleged beneficial effect of isolates without a linear plasmid is only quantitatively demonstrated at the root hair formation level, the accuracy of which can be questioned given the data on GIC26 (Figure 5 in Savory et al., 2017). Root hair formation can be triggered by many factors one of which is growth *on* medium as opposed to *in* medium (Figure 1). Given that the seedlings were grown vertically, this could explain the observed effect. To rule out that the root hair induction is not an artifact of the experimental setup, the percentage of plants showing this effect for all of the treatments has to be provided. The claim that lateral roots developed earlier and more numerous with the nonpathogenic isolates is not supported by quantitative data. The images in the corrected version of Figure 5—figure supplement 1 (Savory et al., 2018) do not suffice to make this point since more control plants have a single secondary root than infected plants. Additionally, the primary root length remains unchanged or is even reduced upon inoculation by all isolates. This is hard to reconciled with a benign effect. Plant development was examined up to two months after inoculation. If the modification of the root system truly has a beneficial effect, the impact on plant development at the end of the experiment must be significant. Indeed, Francis et al. (2016) reported a condition-dependent increase in fresh shoot weight of *Arabidopsis* of 58% after infection with D188-5 at 19 dpi, so data should be provided to substantiate this point in *N. benthamiana*.

## Evidence for growth promotion by and virulence of PBTS isolates

Savory et al. prematurely assume that PBTS-associated *Rhodococcus* isolates have the same mode of action as leafy-gall inducers, although the current knowledge of the PBTS (pistachio bushy top syndrome) species is in its infancy (Stamler et al., 2015a, 2015b, 2016). In contrast, the epidemiology of the leafy gall-inducing *R. fascians* isolates could be understood only because the genomes of 60 isolates were analyzed. It is not possible to draw any definitive conclusions for PBTS based on the analysis of two single isolates.

The conclusion that the PBTS isolates do not cause disease on different hosts, but stimulate root development, is unsupported. For *N. benthamiana*, in Figure 4C, nearly no effect of PBTS1 and PBTS2 is seen on root elongation, yet in Figure 7B and Figure 7—figure supplement 1; panel B there is a significant reduction in root length; Figure 6C is inconclusive because the control is missing. For pistachio, 30 days after inoculation, the plants were only 5 cm tall and none of the plants grew for the last two time points (Figure 7—figure supplement 1; panel E). Given that the rootstock UCB-1 has a very high vigor, these observations indicate that there was something wrong with the plants or the growth conditions.

The remark that the bacterial titer used in previous work was too high is not substantiated by the presented data, unless statistics are provided to show that the observed decrease in root length with increasing titers is significant.

The statement that the molecular detection of the virulence genes was an artifact is based on the fact that the PBTS *fasD* sequences are identical, implying this feature is unusual. However, the *fasD* gene over its entire sequence is identical for most sequenced isolates (Creason et al., 2014) and Savory et al. could not detect any informative SNPs in the linear plasmids.

## Appropriateness of diagnostic tools

Repeatedly, the data reported by Nikolaeva et al. (2009) are rejected by Savory et al. to support the statement that plasmid loss cannot be at the basis for the occurrence of nonpathogenic (PBTS) isolates. Nikolaeva et al. obtained pure isolates – and not populations as stated by Savory et al. – from symptomatic ornamental plants that, when reinoculated as pure cultures on axenically grown peas, had a varying degree of pathogenicity (Nikolaeva et al., 2009). Further, the reisolated bacteria from isolates exhibiting variable pathogenicity now consisted of two subpopulations, one with *fasD* and one without. Importantly, in the context of PBTS, one in three isolates had an unstable virulence replicon and one in three was nonpathogenic. Thus, different genetic lineages cannot be at the basis of presence/absence of virulence genes as suggested. Still, these two processes – plasmid loss and occurrence of different genetic lineages – are not mutually exclusive, a conclusion not considered.

## Interpretation of observed phenotypes

The assay to evaluate attenuation considers differences in gall morphology and variability in the capacity to produce dense galls (Maes et al., 2001). Savory et al. did not analyze these parameters, but merely looked at presence/absence of gall induction, which is not sufficient. Similarly, for seedling infection, the published attenuation assay is based on shoot length (Maes et al., 2001) and not root length as used by Savory et al. It would be more appropriate to use the assays commonly used by others, which would permit comparisons to be made in a more robust manner.

Taking into account the expression data that indicate that AttR has a negative effect on its own expression (Maes et al., 2001), the lack of symptom persistence obtained with D188 +L5::*attR* illustrates that by overexpressing AttR, *att* gene expression is repressed which results in an attenuated phenotype; it does not demonstrate a role for *att* in symptom maintenance.

## Identity of D188-5 strain

In the many experiments done with D188-5, growth defects have never been reported (e.g. Figure 4 in Vereecke et al., 2002), not even by Creason et al. (2014) of the same lab as Savory et al.. Additionally, D188-5 has been successfully used as an acceptor to identify mutations in pFiD188 (Crespi et al., 1992), is able to colonize plants very well (Cornelis et al., 2001), and has clear plant growth-promoting effects (Francis et al., 2016), implying that a putative deletion in D188-5 does not compromise its interaction with the plant. In the attesting growth experiment (Figure 6—figure supplement 2), the start inoculum for D188-5 is lower than that of the two other strains and no error bars are given, so no conclusions can be drawn.

## Fas-cytokinins and leafy galls

Savory et al. challenge the current working model without providing data or an alternative hypothesis by summing up arguments that have been reviewed before by Goethals et al., 2001, but fail to refer to a key paper that likely provides the missing link in the ‘trick-with-the-mix’ model (Radhika et al., 2015). Additionally, the wealth of supporting information is entirely ignored (e.g. Klämbt et al., 1966; Thimann and Sachs, 1966; Rathbone and Hall, 1972; Balázs and Sziráki, 1974; Armstrong et al., 1976; Murai et al., 1980; Crespi et al., 1992; Eason et al., 1996; Stange et al., 1996; Depuydt et al., 2008; 2009; Nikolaeva et al., 2009; Pertry et al., 2009; 2010; Stes et al., 2011; Creason et al., 2014; Radhika et al., 2015).

The fact that 6-benzylaminopurine (BA, which is not produced by *R. fascians*, did not affect the shoot of *N. benthamiana* is worrisome (a photograph should be included in Figure 7C to support this statement), since in a similar experiment I obtained a clear response with *N. tabacum* (Figure 1).

## Misrepresentation of previous work

The messages extracted from various references are absent, wrong or misleading.

- … hosts enrich for members of Rhodococcus because of the beneficial traits … .' The enrichment process is not described and the isolates are not related to *R. fascians*.
- ‘… the use of *vicA* to confirm pathogenic *Rhodococcus* (Stamler et al., 2015a, 2015b).' In both papers, the presence of virulence genes is also evaluated.
- ‘… challenge previous conclusions that *Rhodococcus* isolates lacking virulence genes are causative agents …' Stamler et al. stated that *Rhodococcus* isolates with virulence genes were in part responsible for PBTS.
- The *Rhodococcus* species is introduced as being repeatedly recovered from various plant species to support its mutualistic nature, but the ten citations deal with three plant species only.
- It is stated that the linear plasmid has only three virulence loci and the chromosome only one, but these are the only four loci identified/studied so far.

## Statements about PBTS

No sources are given for the following claims: (a) ‘A second incidence of pistachio bushy top syndrome occurred in 2016’; (b) ‘The misdiagnosis perpetuated the unnecessary removal of trees and exacerbated economic losses’; (c) ‘The detection of *vicA* was used as evidence […] to guide management practices’.
